# Community-based intermittent mass testing and treatment for malaria in an area of high transmission intensity, western Kenya: development of study site infrastructure and lessons learned

**DOI:** 10.1186/s12936-019-2896-6

**Published:** 2019-07-29

**Authors:** Norbert Awino Odero, Aaron M. Samuels, Wycliffe Odongo, Bernard Abong’o, John Gimnig, Kephas Otieno, Christopher Odero, David Obor, Maurice Ombok, Vincent Were, Tony Sang, Mary J. Hamel, S. Patrick Kachur, Laurence Slutsker, Kim A. Lindblade, Simon Kariuki, Meghna Desai

**Affiliations:** 1 Kenya Medical Research Institute (KEMRI), Centre for Global Health Research, Kisumu, Kenya; 2 Centers for Disease Control and Prevention (CDC), Atlanta, GA USA

**Keywords:** Malaria, Elimination, Kenya, MTaT, Infrastructure

## Abstract

**Background:**

Malaria transmission is high in western Kenya and the asymptomatic infected population plays a significant role in driving the transmission. Mathematical modelling and simulation programs suggest that interventions targeting asymptomatic infections through mass testing and treatment (MTaT) or mass drug administration (MDA) have the potential to reduce malaria transmission when combined with existing interventions.

**Objective:**

This paper describes the study site, capacity development efforts required, and lessons learned for implementing a multi-year community-based cluster-randomized controlled trial to evaluate the impact of MTaT for malaria transmission reduction in an area of high transmission in western Kenya.

**Methods:**

The study partnered with Kenya’s Ministry of Health (MOH) and other organizations on community sensitization and engagement to mobilize, train and deploy community health volunteers (CHVs) to deliver MTaT in the community. Within the health facilities, the study availed staff, medical and laboratory supplies and strengthened health information management system to monitor progress and evaluate impact of intervention.

**Results:**

More than 80 Kenya MOH CHVs, 13 clinical officers, field workers, data and logistical staff were trained to carry out MTaT three times a year for 2 years in a population of approximately 90,000 individuals. A supply chain management was adapted to meet daily demands for large volumes of commodities despite the limitation of few MOH facilities having ideal storage conditions. Modern technology was adapted more to meet the needs of the high daily volume of collected data.

**Conclusions:**

In resource-constrained settings, large interventions require capacity building and logistical planning. This study found that investing in relationships with the communities, local governments, and other partners, and identifying and equipping the appropriate staff with the skills and technology to perform tasks are important factors for success in delivering an intervention like MTaT.

## Background

Over the last 15 years, the burden of malaria in many endemic countries has been reduced significantly [1]. In some countries morbidity reduction has led to the transition from control to pre-elimination [2], but in others progress has stagnated. This is likely multifactorial, reflecting ecological factors (e.g. differences in mosquito habitat density, transmission intensity), the innate infrastructure and capacity of the region (e.g. roadways, supply chains, housing conditions), and the quality and coverage of malaria prevention and control measures (e.g. vector control and case management), but there is growing belief that asymptomatic infections, estimated to be > 60% of all prevalent infections even in low transmission areas [3], may contribute to ongoing transmission. Current case management strategies do not reach these individuals, as there are not symptoms to trigger care-seeking and treatment.

Community-based strategies that target all infections, such as mass testing and treatment (MTaT) or mass drug administration (MDA), may lead to transmission reduction and accelerate progress towards malaria elimination [3, 4]. To reduce transmission, high MTaT or MDA coverage is likely necessary as the malaria vector is extremely efficient at transmitting parasites, such that even a small proportion of missed infections may result in rapid rebound to pre-intervention levels [5–7]. Prior experiences with MDA demonstrate that higher population coverage can be obtained through strong community engagement, and by house-to-house visits for delivery of anti-malarials [4, 8]. To achieve malaria elimination goals in resource poor settings, programmes must determine how best to engage and involve the community, identify, train and equip the appropriate staff, manage supply chain logistics, and be flexible enough to adapt strategies to reach as many community members as possible as unexpected challenges arise.

This manuscript describes the study site, community engagement strategies, infrastructure development, and the lessons learned in conducting a cluster-randomized controlled MTaT study in an area of high malaria transmission in western Kenya.

## Description of study site

**Fig. 1:**
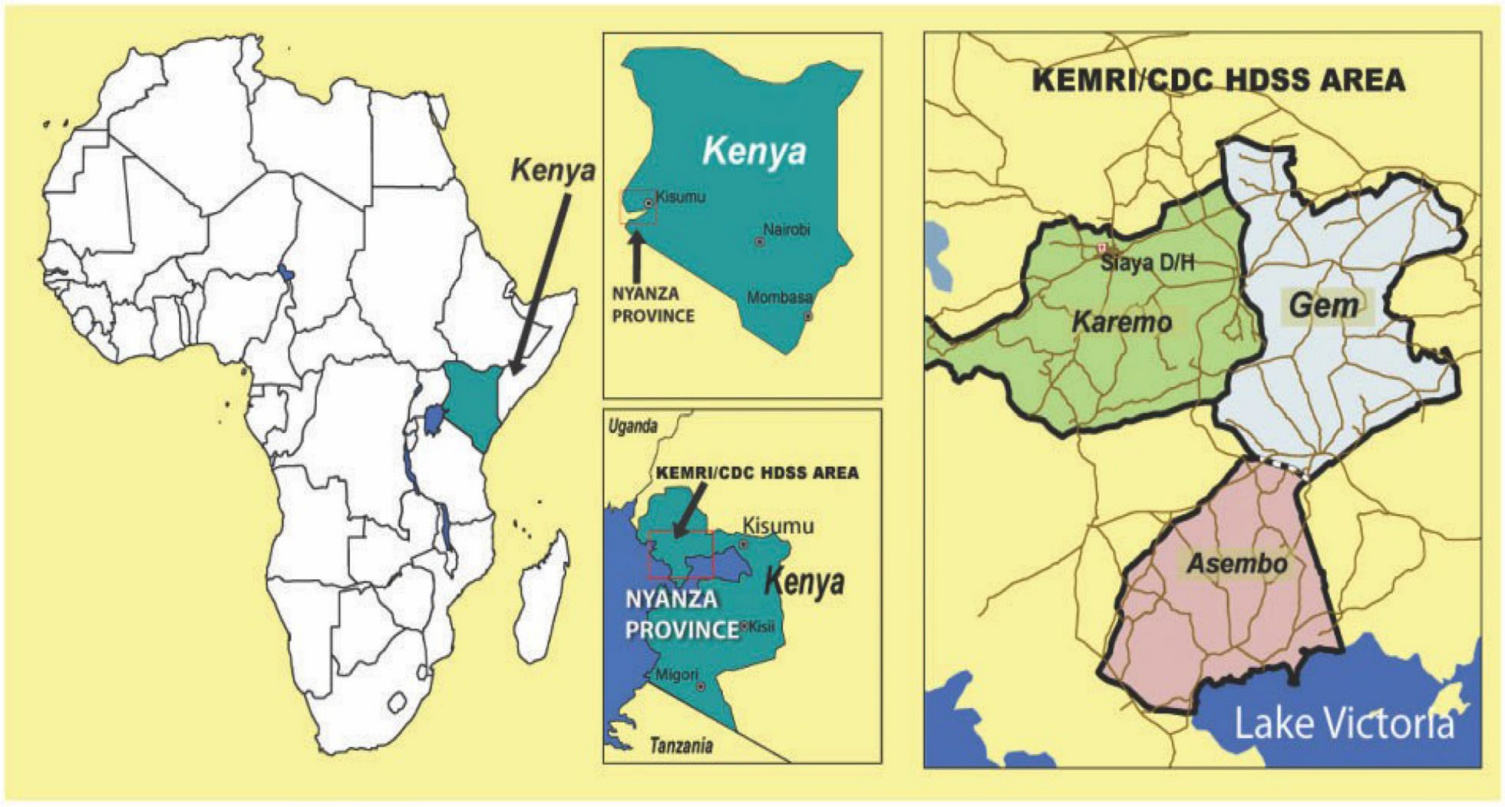
Map of KEMRI-CDC Health and Demographic Surveillance System, Kenya [9]

The study was implemented in Siaya County within the Kenya Medical Research Institute (KEMRI) and US Centers for Disease Control and Prevention (CDC) Health and Demographic Surveillance System (HDSS) (Fig. 1). KEMRI and CDC have been collaborating to conduct research and evaluate malaria control programmes in western Kenya for 40 years [9–11]. During this time, community members have become used to regular requests to enroll in research activities, and many have developed trust with local research organizations and are readily willing to engage in new research and programs [12].

## Population demographic and socio-economic indicators

The HDSS area and study site are described in detail elsewhere [10]. Briefly, the population is mainly rural and comprises of families of the Luo ethnic community who are culturally homogeneous and derive livelihoods from small-scale farming, fishing, and local trading [10]. At study commencement, 61% of the adult population had completed only primary education, 86% of households used firewood for cooking, and > 73% collected water from a source other than a borehole or well (KEMRI and CDC collaboration unpublished data, 2012). Rainfall is seasonal with the heaviest rains occurring between March and May and between October and December. Roads are unpaved and many become impassable by vehicle during these rainy seasons. Average temperatures range between 17 °C and 35 °C. Most of the houses are built of mud, cement or bricks with roofs of corrugated iron sheet or thatched grass. The paths connecting homesteads within and between villages are narrow and overgrown making movement tedious and time consuming. Locating people at homes during the day is particularly difficult as most are engaged in cultivating agricultural lands that lie adjacent to homesteads during the day. The 141 MTaT villages included both urban and rural settlements. The urban areas are small permanent settlements that are clustered mostly around the main roads. Most of the houses in urban centers are rented or leased and do not serve as the permanent or ancestral homes of the MTaT participants. The population living in the study area has low to moderate education levels. In Asembo, for example, only 67.2% of residents aged ≥ 15 years had completed primary education, 17.0% had secondary education or higher, and the remainder (15.8%) had no education in 2002 [10].

## Health statistics and infrastructure

The area is characterized by high mortality rates; the infant and under five mortality rates were 50 and 82 per 1000 live births, respectively, in 2014, and were among the highest in Kenya [13]. Malaria is endemic and transmission occurs throughout the year with two seasonal peaks following the rainy seasons in be June–July and December–January [13]. In 2010, prior to study commencement, nationally representative survey indicated that malaria prevalence in children aged 3 months–14 years by microscopy was approximately 11% nationally and 38% in the lake endemic region (where the study site is situated) [14].

While there are numerous health facilities in the area, many do not have electricity, and are not adequately staffed. Artemisinin-based combination therapy (ACT) was adopted as the first-line for malaria treatment in 2004, and universal testing by microscopy or rapid diagnostic tests (RDTs) was adopted in 2012 [14]. However, the study area is characterized by periodic interruptions in laboratory and pharmaceutical supplies with only 51% of facilities reporting to have lab diagnostic capacity for malaria and only 88% with ACT [15]. In 2013, prior to the start of this study, the Kenya Ministry of Health started implementing integrated Community Case Management (iCCM). In this strategy, community health volunteers (CHVs), a cadre of lay workers, were tasked with visiting all households within communities of residence (villages or a cluster of adjacent villages) and assessing and treating uncomplicated cases of diarrhoea, pneumonia, and malaria. However, iCCM has not been consistently implemented in the MTaT study areas.

## Study procedures

### Design and evaluation methods

A detailed description of the study design and rationale has been published previously [11]. Briefly, this was a community-based, cluster-randomized controlled trial. Ten health facilities were purposively selected, and all 141 villages whose geographic midpoints were located within 3 km of one of the health facilities were included in the study. Three clusters were created around each health facility by merging adjacent villages and then two were randomized to the control and the other to the intervention arm, such that there were approximately 60,000 and 30,000 individuals in the control and intervention arms, respectively. Three times per year for 2 years, study teams visited each household within the intervention communities, tested all consenting individuals with malaria RDTs, and treated all positive individuals with dihydroartemisinin-piperaquine (Eurartesim^®^, Sigman-Tau, Pomezia, Italy). Each round was to be completed within 4–6 weeks. Three strategies were used to evaluate the intervention: (1) comparison of the incidence of clinical malaria through passive case detection at outpatient departments (OPD) of study health facilities, (2) comparison of the incidence of malaria infection measured through a prospective annual cohort tested on a monthly basis at study health facilities; and, (3) comparison of malaria infection prevalence through annual cross-sectional surveys.

### Community engagement and sensitization activities

Prior to initiating study activities, MTaT staff presented an overview of the study to the local administration of study villages. Thereafter, the study was introduced at *barazas* (centralized open community meetings), and community members were provided an opportunity to ask questions and voice concerns to study staff. Community approval meetings were organized with the County Health Management Team (CHMT) through the County Director of Health, education officers and head teachers from the 47 area schools, KEMRI and CDC Community Advisory Board (CAB) members, and village reporters (VRs) and CHVs. The CAB is comprised of community members elected from civil society, community-based organizations and representative of special groups such youth and women groups to represent a number of villages, while VRs are community members recruited by the HDSS to report on vital events such as births and deaths in assigned villages. Prior to study initiation, focus group discussions (FGDs) were held with opinion leaders, CHVs, and community members, segregated by sex, to assess perceptions and potential strategies to improve acceptance [12].

Before each MTaT round, short messages were transmitted during local language radio-spots to raise awareness of the campaigns, and community sensitization activities were continued to obtain feedback from stakeholders to improve study activities. After the first MTaT round, FGDs were repeated in intervention clusters to gather community perceptions and acceptability of the intervention [13]. Information from the FGDs were reviewed and used to improve future rounds. Additionally, regular updates were provided to CHMTs through reports and meetings.

### Mapping and conducting the census

HDSS procedures included updating map and census data of the area three times a year. However, 13 additional villages within the MTaT study area had not been mapped prior to the study start; these villages were mapped per HDSS procedures [10] using handheld devices, including personal digital assistants (PDAs) and tablets, with built-in geographical positioning systems (GPS) to capture accurate coordinates and to store data in the field. Additionally, at each MTaT, cross-sectional, and HDSS mapping rounds, compounds and houses that were newly constructed or that had been abandoned or destroyed, and household rosters, incorporating births, deaths, and migrations, were updated on maps and census databases that were shared for harmonization with the overall HDSS data management system. Accurate mapping and census data were used to identify households and quantify the population denominator for coverage calculations.

## Study implementation staffing requirements and training

### Staffing overview and categorization

**Fig. 2:**
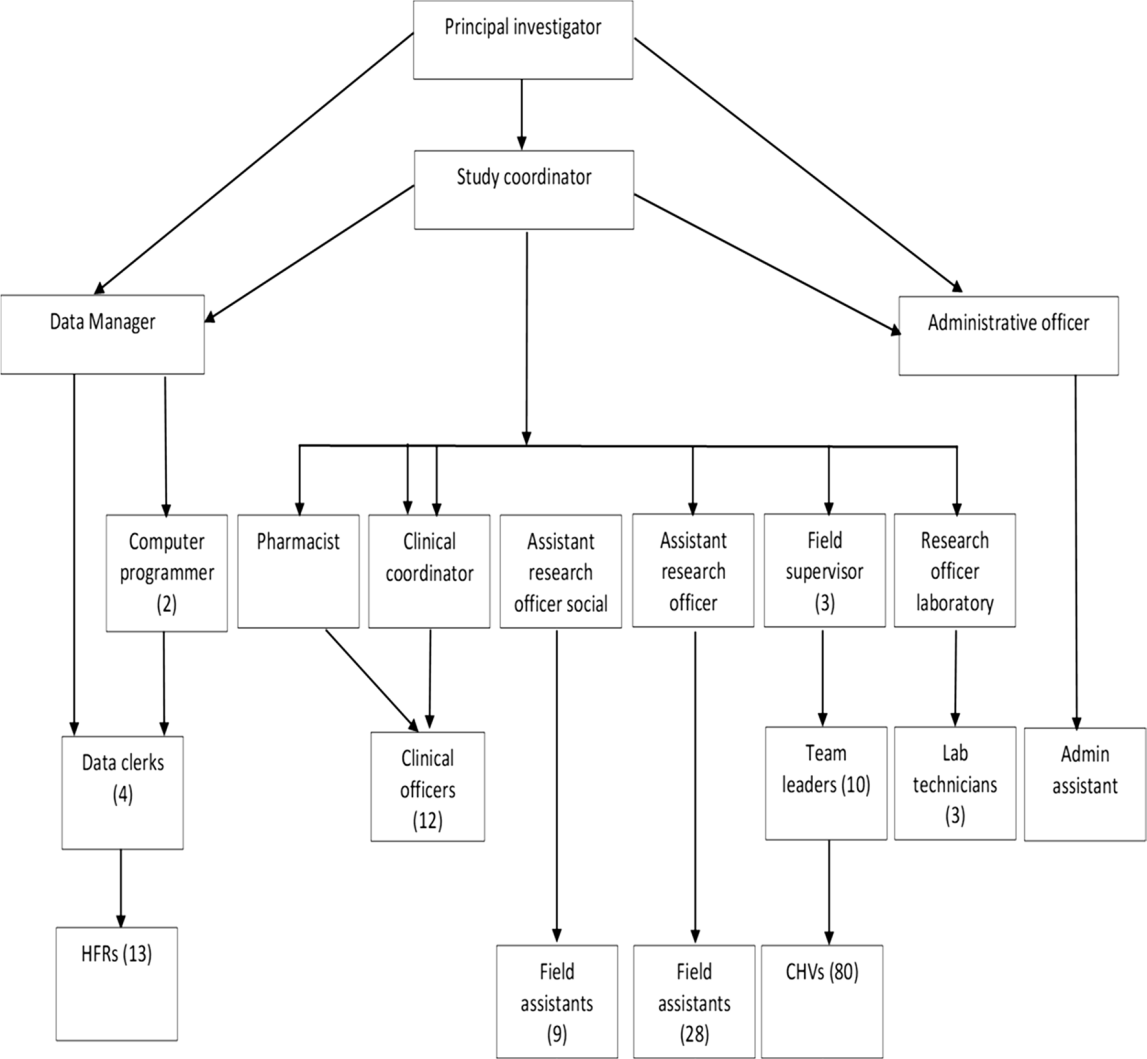
Diagrammatic representation of supervisory and staffing structure for MTaT

A large study team (Fig. 2) of multiple cadres was hired, rigorously and repeatedly trained, and supervised. An overall study coordinator provided supervision, oversight, and coordination to leads of each of the study sections: data management, clinical care, social sciences, field activities, laboratory, and administration/logistics; these leads in turn provided oversight to a cadre of staff, in addition to specific duties described in the following sections. For ease of oversight, teams were grouped into Field, Health Facilities, Data, and Logistics Sections.

### Field section responsibilities

The field section was responsible for the implementation of cross-sectional surveys and MTaT rounds, and was comprised of 3 field supervisors, 10 team leads, and 80 CHVs and/or field workers (FWs). Field supervisors, team leaders and FWs are cadres of staff with prior experience performing community-based studies within the HDSS, with the higher ranking staff having more experience. Each field supervisor was responsible for the overall conduct of study activities in a geographical area within the HDSS (Fig. 1). Field supervisors provided supervision to 3–4 team leaders and met with the CHVs and (FWs) as a group at the end of each day to review work completed and address any emergent issues. Team leaders provided oversight and direction to approximately 9 FWs or CHVs during MTaT and cross-sectional rounds, meeting with team members each morning to assign households to visit that day, give feedback, and discuss challenges. Additionally, team leaders provided supplies to, and received samples from CHVs and FWs daily, and transported these by motorcycle to and from study health facilities. Initially, when PDAs were used for data collection, team leaders were responsible for transporting the PDAs from the data center to the CHVs and FWs every morning, and back to the data center for download every evening. Lab technicians were responsible for ongoing mentorship of CHVs and FWs to ensure quality sample collection.

During MTaT and cross-sectional rounds, CHVs and FWs were paired to visit houses and administer questionnaires, test for malaria by RDT and for pregnancy when appropriate, collect dried blood spots (DBS) on filter paper, and provide treatment and referral per study algorithms. During the first round of MTaT, or when encountering new individuals, the CHVs and FWs obtained informed consent for study procedures for the current and subsequent rounds.

### Health facilities

**Fig. 3:**
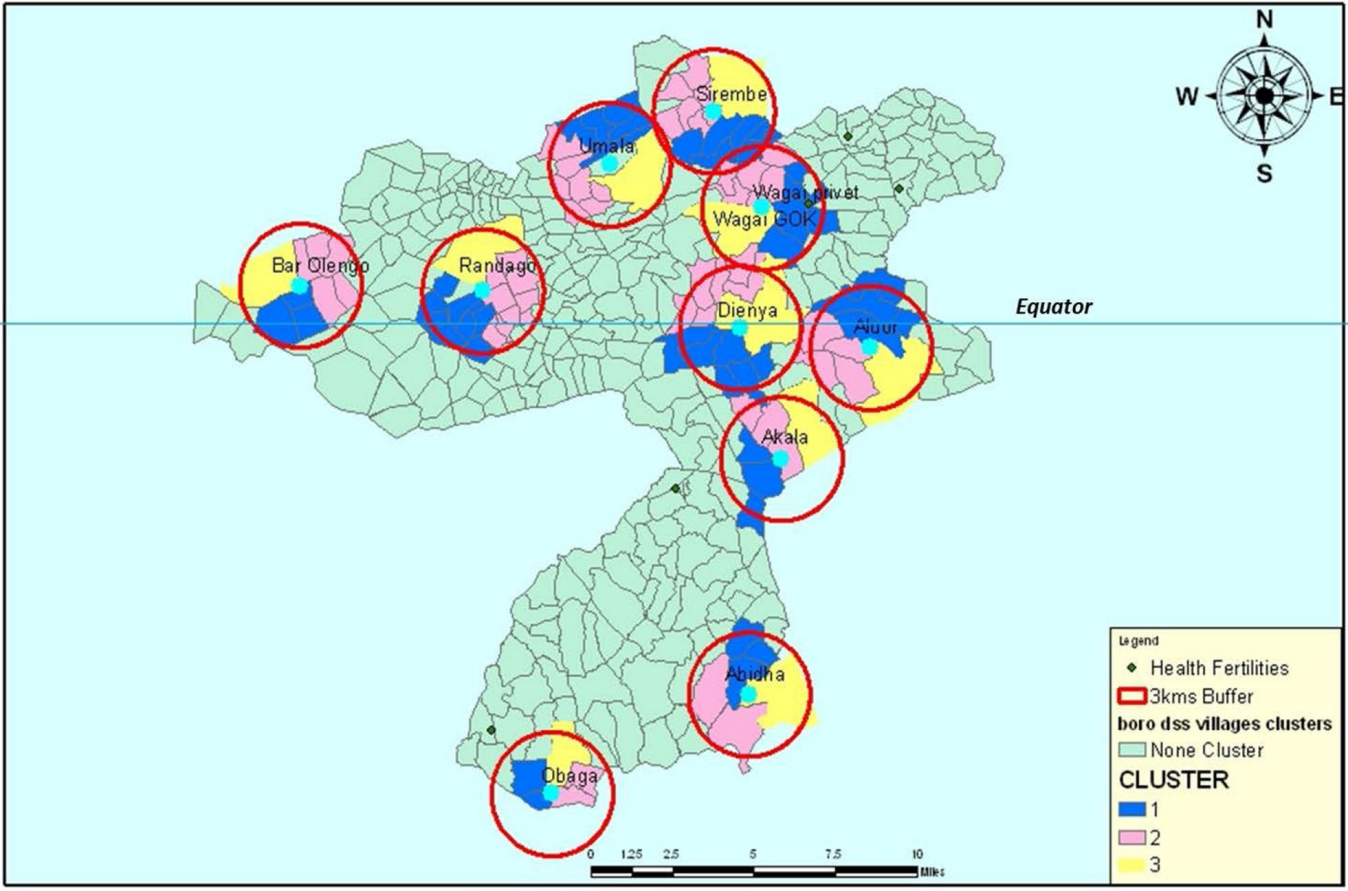
Map showing the MTAT health facilities in the study catchment area

Public health facilities are staffed by Kenya Ministry of Health (MOH) staff of various cadres, including clerks, who record patients’ demographic characteristics, vital signs, diagnosis and treatment; and clinical officers (COs) and nursing officers (NOs) who provide clinical management. Based on prior experience, the study team was aware that MOH staff become overwhelmed with large volumes of patients, and do not always have adequate time to consistently perform procedures per research study protocols. To ensure high quality data to evaluate the efficacy of MTaT, the study team established a presence in all 10 study health facilities (Fig. 3), and hired study-specific staff to conduct passive and active case detection. After memoranda of agreement were established with CHMTs, 13 health facility clerks and 12 COs were recruited, trained and deployed at the 10 study health facilities.

Health facility clerks were directly supervised by data clerks, who regularly visited health facilities to download data, and ensure quality assurance of the data. Health facility clerks used netbooks containing a database of registered HDSS residents. For each individual attending the OPD of study health facilities, health facility clerks queried the database using the individual’s HDSS ID to determine if the individual resided within the study catchment area. Data from health facility visits for all individuals residing within the study catchment area were entered into a database on the netbooks. Data collected included age, gender, presenting symptoms, presence or history of fever, tests performed and results, diagnoses made, and treatments given.

A lead CO provided supervision, expert consultation, quality control of data from visits, and back-up support for COs while they managed cohort participants. Cohort members were instructed to visit the study health facility closest to places of residence on a monthly basis for scheduled visits, regardless of whether they had any symptoms, and if they felt ill at any time for sick visits (unscheduled visits). Study-specific COs performed medical histories and physical exams at each cohort visit, administered a study questionnaire, and collected blood for preparation of a malaria blood smear, RDT, dried blood spots (DBS) on filter paper, and hemoglobin levels.

### Data management

The data team consisted of a lead data manager who oversaw two programmers, and four data clerks who were responsible for verifying, uploading and cleaning data, raising queries to field teams, and responding to data requests from staff. Data programmers were responsible for programming the questionnaires used in MTaT rounds, cross-sectional surveys, and passive and active case detection into electronic surveys to be used on PDAs, tablets, and netbooks. The permanent data center at the KEMRI and CDC main office was adapted to accommodate MTaT databases, and a temporary hub was established at Siaya County Referral Hospital. During the field activities, the temporary data centre served as the point of downloading and cleaning data, and charging handheld computers and tablets. Data staff were responsible for reviewing the collected data each night to inform the team leads which houses needed to be visited the next day to optimize data completeness. Data clerks periodically visited study health facilities to download data from the netbooks and PDAs used by the Health Facility Clerks and the COs, respectively, and transferred these data to the temporary and eventually primary hub.

Initially, survey questionnaires were programmed into netbooks and PDAs. At the end of each day, PDAs were returned to the temporary data hub, and data were downloaded serially from each of the 40 PDAs via 4 computers to the secured databases. The PDAs presented three distinct problems: (1) they were not network enabled and had to be brought to a central location for download, (2) data from PDAs had to be serially downloaded to computers—it took approximately 4–6 h each night to download data from the 40 PDAs to the 4 study computers, and (3) when PDAs were damaged or destroyed. It was also difficult to find replacements since production of this model had been discontinued by the manufacturer. To address these issues, the study switched from PDAs to handheld ASUS Google Nexus 7 tablets (^©^ASUSTeK Computer Inc., US). Study programmers learned to program in Open Data Kit^®^ (ODK) software [16], which allowed for transmission of data over Wi-Fi/3G networks to a secure study-specific cloud server via secure virtual private network (VPN), and simultaneous download from 40 tablets. Data on all study devices were encrypted, and a triple backup system was set up on both the local and cloud servers allowing for restricted, remote, and simultaneous access.

### Logistics

The study logistics were overseen by an administrative officer and 2 administrative assistants who worked together to purchase, store, and transport study supplies on a regular basis. Bulk supplies (e.g. 20,000 LLINs, 180,000 RDTs, 90,000 doses of ACT) were purchased by the President’s Malaria Initiative (PMI) and received through the KEMRI and CDC procurement system in Kisumu, and transported to the KEMRI and CDC Clinical Research Center (CRC) in Kisumu town on a weekly basis. From the CRC, supplies were transported to temporary stores at health facilities, where team leaders would distribute supplies daily, or as necessary, to the CHVs and FWs.

### Training for MTaT implementation

Prior to each MTaT round, approximately 100 field staff were trained through classroom presentations, individual mentorship and hands-on practical sessions, including 2 days of piloting. The initial training lasted 7 days, but subsequent trainings were shortened to 5 days. At the end of the training activities, supervisors evaluated CHVs and FWs for competency in performance of study procedures, including informed consent, using tablets to complete questionnaires, performing blood draws, and following treatment and referral algorithms. At the completion of the training, 80 staff were chosen based on performance. In selecting staff, preference was given to CHVs over FWs as an intention of the study was to utilize and build capacity of MOH staff.

COs implementing active surveillance were trained on malaria treatment algorithms, and study-specific procedures. In addition, study COs attended additional training on malaria case management sponsored by local implementing partners. All study staff were trained on the MTaT protocol, standard operating procedures (SOPs) and good clinical practices (GCP).

## Cost-efficiencies

Throughout the study, expenditures related to the preparation and implementation of MTaT rounds were computed (Table 1). Costs were categorized as personnel, transport, training, community mobilization, communication and supplies. The change in cost from one round to the subsequent round, and from the first round to the final round, was expressed as a percentage change; positive percentages indicate savings, while negative percentages indicate an increase in cost from one round to the next. While there were realized savings in all areas except in community mobilization, the largest absolute and relative gains were in transportation, supplies, and personnel. Overall, from Round 1 to Round 6, 38.6% cost-savings was realized.

**Table 1:** Costs and % change in costs per Items from round 1 to 6 of MTaT

Cost items (in USD)	R1 cost USD	R2 cost USD	% (R1–R2)^a^	R3 cost USD	% (R2–R3)	R4 cost USD	% (R3–R4)	R5 cost USD	% (R4–R5)	R6 cost USD	% (R5–R6)	% (R1–R6)
Personnel	61,936	57,053	7.90	52,667	7.70	59,504	− 13.00	59,504	0.00	52,667	11.50	15.00
Transport	23,625	22,275	5.70	23,250	− 4.40	9231	60.30	8444	8.50	4875	42.30	79.40
Trainings	15,600	18,000	− 15.40	11,700	35.00	11,700	0.00	3000	74.40	7694	− 156.50	50.70
Community mobilization	2190	2190	0.00	2190	0.00	2190	0.00	2190	0.00	2190	0.00	0.00
Supplies (drugs, shipment, ITNS etc.)	15,000	10,000	33.30	10,000	0.00	5000	50.00	5000	0.00	5,000	0.00	66.70
Communications	1000	1000	0.00	1000	0.00	1000	0.00	1000	0.00	875	12.50	12.50
Total	119,351	110,518	7.4	100,807	8.8	88,625	12.1	79,138	10.7	73,301	7.4	38.6

^a^Positive percentage difference represents savings, negative represents increased costs

## Discussion

Study team developed infrastructure to deliver and evaluate a population-based intervention to approximately 30,000 residents of western Kenya three times a year for 2 years. Throughout the course of the study, approaches were adapted either to improve efficiencies from lessons learned, or as a result of unforeseeable or uncontrollable events.

### It is valuable to understand locally available human resources and adapt to them

In considering an intervention of this scale, identifying the right personnel and determining how best to provide support proved to be critical to the study. It was anticipated that working with CHVs would improve the acceptance of the intervention as CHVs were well respected within communities of residence. An example of how the continued trust between the CHVs and the community led to intervention acceptance included convincing asymptomatic people who tested positive for malaria to take and complete a course of ACT. The trust was further evident in the successful conduct of pregnancy testing of women of child bearing age who tested positive for malaria. It was also anticipated that by building capacity in this cadre, the study would assist in improving future community care. Additionally, the use of CHVs was operationally feasible and cost effective. However, iCCM had been adopted in western Kenya a few months before study commencement and CHVs were still largely inexperienced with malaria testing and treatment activities. The study team decided to begin working with a mixture of trained, experienced FWs and CHVs.

Prior to working with MTaT, the CHVs had received trainings on how to perform RDTs and provide malaria treatment. The CHVs struggled to perform RDTs during the training and pilot sessions. From direct feedback and observations, it was noted that trainings were far more successful when they were iterative, hands-on, and delivered in the local Luo language. Trainings were adapted to include more individual piloting, and role-playing with direct supervision. The perceived negative of including FWs into the study proved advantageous as FWs provided hands-on training to the CHVs during the trainings, and when paired with them during MTaT rounds.

When administering malaria treatment, decisions need to be made in the choice of anti-malarial based on age, pregnancy status, and history of adverse event or drug reaction. To assist the CHVs, study data team created and programmed a step-by-step diagnostic and treatment algorithm into the PDAs and tablets in the local language instructing when to perform pregnancy testing, to probe for a history of drug reactions, and then indicated which drug to prescribe and when to recommend referral to a health facility. The algorithm had dual benefit; it greatly reduced the risk of incorrect treatments and related adverse events, and saved time by assisting CHVs through the decision-making tree. This was particularly helpful in the context of CHV turn-overs experienced by the study as a result of other competing priorities of the CHVs (e.g. catch up immunization activities).

By the third round, MTaT study no longer required the assistance of FWs, and successively increased coverage to above 90%. One possible explanation for the increase in coverage was because CHVs were able to use community relationships and knowledge to track the hard-to-reach residents, such as farmers, motorcycle operators, and school-going children.

### Know staffing needs and the environment they work in

In round one of MTaT, Team Leaders had not been introduced, the study relied on 3 Field Supervisors to provide support and supplies to the 40 CHV/FW teams every day. This proved to be overwhelming as teams were spread across long distances, and the road infrastructure was poor and motor vehicles could not always pass through narrow or muddy roads. In round 2, the study recruited 10 team leaders, who provided extra level of support below the field supervisors, and motor vehicles were also replaced with motorcycles that could access remote areas in the community more easily. In addition, Supervisors were provided with netbooks uploaded with the MTaT database to assist in assigning study compounds to study teams, and monitoring the number of compounds completed. This provided more direction and supervision to the CHVs and FWs, which resulted in efficiencies such that while the first MTaT round took 6 weeks to complete, subsequent rounds were completed in 4 weeks. And, despite the increased staffing and motorbikes, these gained efficiencies manifested in a realized cost-savings of an overall 38.6% savings gained between rounds 1 and 6 (Table 1).

### Invest in technology

At the peak of the rounds, study teams were delivering the intervention to approximately 1000 study participants per day generating a tremendous amount of data to be managed daily. This, coupled with the initial system of using PDAs, overwhelmed study data team. The transition to tablets benefitted the CHVs as tablets had a longer battery life, better resolution, and a wider screen which made it easier to maneuver throughout the programmed survey and algorithm. Additionally, the tablets were enabled with more accurate global positioning systems, which aided in finding households, and the camera operated as a scanner for sample bar codes, which saved time and reduced errors in manual entry of sample codes. Perhaps the greatest gain from the tablets was the ability to transmit data from the field through a cloud server. This reduced the daily transport needs of the team leaders and field supervisors, and saved 4–6 h daily for the data management staff, which allowed the data staff more time to focus on data cleaning and queries.

### Logistics for storage and transport

Conducting a large field trial with a massive amount of supplies would have been logistically impossible without a decentralized storage system. Inadequate space for storage of large volumes of study supplies coupled with warm (> 30 °C) daytime temperatures exceeding storage recommendations for the RDTs and ACT further complicated supply management. Study team learned to stagger the procurement and delivery of commodities according to the demands and available space at the study health facilities. Though this required a higher level of logistical coordination, it enabled efficient supply of commodities.

### Expect the unexpected, collaborate with in-country partners, and engage the community

Kenya’s new constitution was implemented during the 2nd year of MTaT which affected study activities such as the introduction of a new structure in health care management, an industrial strike by the MOH clinical staff, and new taxes such as the Railway Development Levy (1.5% of custom value of goods). This delayed the clearance of health commodities from the port of Mombasa resulting in stock-outs and unanticipated costs to the study. The study benefited from actions of partners such as US government funded (PMI), which provided assistance in accelerating the clearance of essential health commodities (Table 2).

**Table 2:** Thematic summary of challenges in respective areas of study activities

Field based	Poor road networks, unreliable electricity supply Sparse spread out populations Late completion of field work activities and late arrival of samples in the lab Inadequate storage facilities and large volume of supplies
Health facility based	Periodic stock outs of ALs and RDTs Non adherence to MOH malaria management algorithms Charging user-fees for RDT diagnosis Inadequate office space for use by the study Slow or non-response by the DHMTs on various issues impacting the study Lack of proper records of stocks of RDTs and ACTs
Technical	Old handheld computers with slow charging, poor power retention and breakdowns Lengthy and arduous data reconciliation process due to wrong handheld computer entries Manual download of handheld computers needing staff presence overnight Long periods of training Long and complex training strategies
Political	Industrial strike by MOH staff Enactment of new constitution with introduction of new taxes

The ability to effectively work with the MOH clinicians and county health management teams to develop reporting systems to forecast stock-outs greatly improved clinical care and reduced cases of stock outs. Continuous engagement with the community through VRs and CAB meetings ensured feedback and provided opportunity to promptly address emerging issues. Strong relationships with other non-health sectors such as the local administration and schools ensured that the MTaT study was widely accepted and owned by the local community resulting in high intervention coverage.

### Can a malaria control programme feasibly implement and monitor MTaT?

In considering MTaT or MDA in other areas, one must consider benefits that were afforded to the study from using HDSS, and experienced research staff to organize and mobilize activities. If this study had been implemented in an area where similar capacity did not exist, substantial input would be necessary. Conversely, this was developed as a study to gather high quality data to test hypotheses, and if implemented as a programme, many of the challenges faced might not exist.

The experience with MTAT illustrated invaluable benefits that local health facilities provide. By setting up temporary field offices in the 10 local health facilities and working very closely with local health facilities management, the study was able to assign CHVs to facilities closest to villages they were residing in and familiar with. This strategy reduced time spent locating participants’ compounds. Therefore, in a program setting, implementation of MTaT could leverage support from local health facilities within catchment that could act as hubs for localized support (e.g. provision of storage space and distribution of supply, provision of supervisory structure for CHVs and collection of process and impact indicators).

If MTaT is implemented as a malaria control programme, there would not be a need for informed consents, survey questionnaires, and the collection of dried blood spots, all of which increased the need for training, skilled staff, and time to complete the required procedures. Infrastructure under local MOH such human resources at local health facilities would be useful in delivering strategies to improve MTaT coverage.

While CHVs delivering MTaT as a programme would not require the level of supervisory oversight similar to research settings, the need for effective periodic feedback between CHVs and supervisors at respective health facilities cannot be underestimated. It was learned that through sharing of experiences at the end of in the field, CHVs learned from each other and from supervisors and devised strategies to overcome challenges in the field such as tracking hard-to-reach populations. For example, rather than making random household visits, CHVs worked with health facility staff to conduct mobilization during designated antenatal and postnatal clinic days at the health facilities and set up appointments for household visits by CHVs. Study teams reported that participants were easily tracked in households where individual household members had agreed to appointments at the health facility level thus saving time and increasing MTaT coverage.

In the context of routine implementation, monitoring and evaluation of MTaT can rely on routine surveys and health management information system data, such as malaria indicator survey and district health information system 2, respectively, rather than the rigorous requirements of study specific protocol (e.g. informed consents, survey questionnaires, and the collection of dried blood spots) which increase the need for training, skilled staff, and time to complete an encounter.

Further, programme messages can be delivered through health facilities ensuring continuous community engagement to achieve high level of participation and subsequent high coverage of the target population. With appropriate political will, community engagement, strong partnerships at the local health facilities closer to the community, adequate human resources, availability of commodities and dedication to high coverage, MTaT or MDA can be effectively performed by a malaria control programme.

## Conclusion

Conducting a study or intervention of such scale requires a tremendous amount of planning, support, and flexibility. The study team also learned that the key elements were investing in the relationships with the communities, local and national governments, and other partners in the area, as well as identifying, training, and equipping the staff with what they need to succeed. While the study benefited greatly from the existing infrastructure that had been developed by the KEMRI and CDC collaboration over decades, that the intervention was conducted as a study rather than a programme introduced complexities specific to research that that may not exist or may be different should MOH choose to implement it as a routine strategy.

## Data Availability

Not applicable.

## Abbreviations

ACT: artemisinin-based combination therapy
CDC: Centers for Disease Control and Prevention
COs: clinical officers
CRC: Clinical Research Centre
CAB: Community Advisory Board
CHVs: community health volunteers
CHMT: county health management team
DBS: dried blood spots
FWs: field workers
FGDs: focus group discussions
GPS: geographical positioning system
GCP: good clinical practices
HDSS: Health and Demographic Surveillance System
iCCM: integrated Community Case Management
KEMRI: Kenya Medical Research Institute
MDA: Mass Drug Administration
MTaT: mass testing and treatment
MOH: Ministry of Health
NOs: Nursing Officers
ODK: Open Data Kit^®^
OPD: Out-Patient Department
PDAs: personal digital assistants
PMI: President’s Malaria Initiative
RDTs: rapid diagnostic tests
SOPs: standard operating procedures
VRs: village reporters
VPN: virtual private network
